# Secular trends of antimicrobial resistance of blood isolates in a newly founded Greek hospital

**DOI:** 10.1186/1471-2334-6-99

**Published:** 2006-06-15

**Authors:** Matthew E Falagas, Sofia K Kasiakou, Dimitra Nikita, Panayiota Morfou, George Georgoulias, Petros I Rafailidis

**Affiliations:** 1 Alfa Institute of Biomedical Sciences (AIBS), Athens, Greece; 2 Department of Medicine, "Henry Dunant" Hospital, Athens, Greece; 3 Department of Medicine, Tufts University School of Medicine, Boston, Massachusetts, USA; 4 Department of Microbiology, "Henry Dunant" Hospital, Athens, Greece

## Abstract

**Background:**

Antimicrobial resistance is one of the most challenging issues in modern medicine.

**Methods:**

We evaluated the secular trends of the relative frequency of blood isolates and of the pattern of their in vitro antimicrobial susceptibility in our hospital during the last four and a half years.

**Results:**

Overall, the data regarding the relative frequency of blood isolates in our newly founded hospital do not differ significantly from those of hospitals that are functioning for a much longer period of time. A noteworthy emerging problem is the increasing antimicrobial resistance of Gram-negative bacteria, mainly *Acinetobacter baumannii *and *Klebsiella pneumoniae *to various classes of antibiotics. *Acinetobacter baumannii *isolates showed an increase of resistance to amikacin (p = 0.019), ciprofloxacin (p = 0.001), imipenem (p < 0.001), and piperacillin/tazobactam (p = 0.01) between the first and second period of the study.

**Conclusion:**

An alarming increase of the antimicrobial resistance of *Acinetobacter baumannii *isolates has been noted during our study.

## Background

Increasing antimicrobial resistance among bloodstream isolates is considered a significant problem worldwide [1,2]. This is especially true in some areas including the countries of Southern Europe where a considerable proportion of pathogens are resistant to antibiotics of several classes [3]. Although antimicrobial resistance is noted in all pathogens, some phenotypes of resistance such as methicillin resistant *Staphylococcus aureus *(MRSA), vancomycin resistant enterococci (VRE), methicillin resistant coagulase negative staphylococci (MRCNS), and carbapenem resistant enterobacteriacae, *Pseudomonas aeruginosa*, and *Acinetobacter baumannii *are of particular concern. We sought to study the secular trends of the relative frequency and antimicrobial resistance of blood isolates in a newly founded hospital in Greece.

## Methods

### Patient population

The patient population comprised of patients admitted to Henry Dunant Hospital, Athens, Greece in the period of 01/01/2001–30/06/2005. Henry Dunant Hospital was founded in October 2000. It is a general tertiary hospital with 450 beds covering most medical specialties with the exception of pediatrics, obstetrics, and transplant surgery. It has 3 combined medical and surgical intensive care units with a total of 38 beds.

### Microbiological studies

Identification of the microorganisms to the species level was performed with the automated system Vitek 2 (Biomérieux) according to the manufacturer's instructions. Not all, but only the first blood isolate per patient was included in the study. The Bactec system (Becton-Dickinson) was used during 2001, 2002, and 2003, and the BacT Alert 3D (Biomérieux) was used during 2004 and 2005. Isolation of bacteria was followed by susceptibility testing that was performed with the Vitek 2 system, applying the criteria suggested by the Clinical and Laboratory Standards Institute (CLSI) [4,5]. The identification and antimicrobial susceptibility of viridans Streptococci was preformed by the use of API (BioMérieux) and the use of the Kirby-Bauer method. Fungi were identified with the use of the specific card for the Vitek 2 system. Susceptibility to colistin was tested by the Vitek method and the E-test. Pulsed field gel electrophoresis and ribotyping were not performed to exclude secondary outbreak strains.

### Statistical analysis

Differences in proportions were compared by x^2 ^test or Fisher's exact test where appropriate. Statistical significance was set for p < 0.05. All statistical analyses were performed using SPSS 11.0 and S-PLUS 6.1 Professional.

## Results

The frequency of isolation of bacteria from cultures of blood specimens was 182 per 12,593 admissions during 2001 (14.4 per 1,000 admissions), 507 per 25,865 admissions during 2002 (19.6 per 1,000 admissions), 693 per 30,597 admissions during 2003 (22.6 per 1,000 admissions), 566 per 30,599 admissions during 2004 (18.4 per 1,000 admissions) and 208 per 15,683 admissions during 2005 (13.2 per 1,000 admissions). There was a significant difference in the proportion of isolates identified over the 5year period (p < 0.001). The percentage of positive blood cultures to the total number of blood cultures was 4.84% for the year 2001, 6.46% for 2002, 7.91% for 2003, 6.71% for 2004 and 5.7% for 2005. Regarding the relative frequency of the bacteria isolated from blood specimens, Gram-positive bacteria were more common than Gram-negative bacteria throughout the study period (Table 1). Coagulase negative staphylococci were the commonest blood isolates (52.5 % of total). The relative frequency of other Gram-positive and Gram-negative microorganisms was the following, in descending order: *Escherichia coli *(8.9 %), *Staphylococcus aureus *(5.9 %), *Pseudomonas aeruginosa *(5.2 %), *Klebsiella spp *(4.8 %), *Acinetobacter baumannii *(4.1 %), *Enterococcus faecalis *(2.2 %), and *Enterococcus faecium *(1.8 %). In Table 2 we summarized the relative frequency of blood isolates by service, namely wards and intensive care unit.

**Table 1:** Relative frequency of blood isolates in Henry Dunant Hospital, Athens, Greece (01/01/2001–30/6/2005).

	**Number of isolates (proportion within the year)**						
***Microorganisms***	**2001 (n = 182)**	**2002 (n = 506)**	**2003 (n = 693)**	**2004 (n = 566)**	2005* (n = 208)	**Total**	**p-value**

***Gram-positive***							
*Coagulase-negative staphylococci*	84 (46.2)	282 (55.6)	377 (54.4)	297 (52.5)	94 (45.1)	1134 (52.5)	0.03
Staphylococcus aureus	12 (6.6)	40 (7.9)	42 (6.1)	27 (4.8)	8 (3.8)	129 (5.9)	0.16
*Enterococcus faecalis*	2 (1.1)	15 (3.0)	21 (3.0)	6 (1.1)	4 (1.9)	48 (2.2)	0.09
*Enterococcus faecium*	4 (2.2)	14 (2.8)	8 (1.2)	11 (1.9)	2 (0.9)	39 (1.8)	0.25
***Gram negative***							
Escherichia coli	15 (8.2)	36 (7.1)	57 (8.2)	54 (9.5)	30 (14.4)	192 (8.9)	0.03
Pseudomonas aeruginosa	13 (7.1)	29 (5.7)	35 (5.0)	26 (4.6)	11 (5.3)	114 (5.2)	0.72
Acinetobacter baumannii	10 (5.5)	10 (2.0)	15 (2.2)	38 (6.7)	17 (8.2)	90 (4.1)	<0.001
Proteus mirabilis	3 (1.7)	1 (0.2)	1 (0.1)	4 (0.7)	3 (1.4)	12 (0.5)	0.03
Klebsiella spp.	5 (2.8)	17 (3.4)	39 (5.6)	33 (5.7)	11 (5.3)	105 (4.8)	0.18
Enterobacter spp.	4 (2.2)	8 (1.6)	24 (3.5)	9 (1.6)	3 (1.4)	48 (2.2)	0.11
Salmonella spp.	2 (1.1)	1 (0.2)	4 (0.6)	9 (1.6)	0 (0)	16 (0.7)	0.05
***Others ***(include other Gram positive and Gram negative)	22 (12)	25 (4.9)	34 (4.9)	26 (4.6)	15 (7.2)	122 (5.6)	0.002
***Fungi***							
*Candida*	6 (3.3)	29 (5.7)	36 (5.2)	26 (4.6)	10 (4.8)	107 (4.9)	0.74

**Table 2:** Relative frequency of blood isolates by service in Henry Dunant Hospital, Athens, Greece (01/01/2001–30/6/2005).

	**Number of isolates by service/total number of same species isolates within the year**							
***Microorganisms***	**2002**		**2003**		**2004**		2005*	

***Gram-positive***	Wards	ICU	Wards	ICU	Wards	ICU	Wards	ICU
*Coagulase-negative staphylococci*	158/282	124/282	191/377	186/377	159/297	138/297	49/94	45/94
Staphylococcus aureus	26/40	14/40	21/42	21/42	17/27	10/27	6/8	2/8
*Enterococcus faecalis*	10/15	5/15	13/21	8/21	3/6	3/6	3/4	1/4
*Enterococcus faecium*	10/14	4/14	6//8	2/8	8/11	3/11	2/2	0/2
***Gram negative***								
Escherichia coli	27/36	9/36	48/57	9/57	49/54	5/54	28/30	2/30
Pseudomonas aeruginosa	17/29	12/29	18/35	17/35	14/26	12/26	6/11	5/11
Acinetobacter baumannii	2/10	8/10	7/15	8/15	9/38	29/38	7/17	10/17
Klebsiella spp.	12/17	5/17	25/39	14/39	14/33	19/33	4/11	7/11

We compared the antimicrobial resistance of blood isolates of two periods: the first period was 1/1/2002–31/12/2003 and the second period 1/1/2004–30/6/2005. The year 2001 was not included in the comparison of the antimicrobial resistance because the in vitro susceptibility data were not readily available. The antimicrobial resistance of Gram-negative bacteria isolated from blood in our hospital showed some interesting trends (Table 3). In Table 4 we present data regarding the in vitro susceptibility patterns and the respective MIC_90 _of the isolated bacteria. *Acinetobacter baumannii *isolates showed an increase of resistance to amikacin (p = 0.019), ciprofloxacin (p = 0.001), imipenem (p < 0.001), and piperacillin/tazobactam(p = 0.01) between the first and second period of the study. In addition, we noted the appearance of resistance to polymyxins in one *Acinetobacter baumannii *isolate. Regarding the secular changes of the antimicrobial resistance of *Pseudomonas aeruginosa *isolates during our study, there was only one statistically significant association, namely increased resistance to ceftazidime (p = 0.016).

**Table 3:** Trends of antimicrobial resistance of Gram-positive and Gram-negative bacteria.

	**Number of resistant isolates/total isolates tested (proportion) within the year**						
***Microorganisms***	**2002**	**2003**	**2004**	**2 005**	**2002/2003**	**2004/2005**	p-value**

***Gram-positive***							
***Staphylococcus epidermidis***							
Oxacillin	171/202 (84.7)	215/261 (82.4)	179/219 (81.2)	75/94 (79.8)	386/463 (83.3%)	254/313 (81.2%)	0.10
Gentamicin	144/202 (71.3)	168/261 (64.4)	148/219 (67.6)	54/94 (57.5)	312/463 (67.4%)	202/313 (64.5%)	0.37
Vancomycin	0/202 (0)	0/261 (0)	0/219 (0)	0/94 (0)	0/463 (0%)	0/313 (0%)	NA
***Staphylococcus aureus***							
Oxacillin	25/38 (65.8)	21/30 (70)	12/25 (48)	6/8 (75)	46/68 (67.6%)	18/33 (54.5%)	0.19
Gentamicin	23/38 (60.5)	4/30 (13.3)	6/25(24)	3/8 (37.5)	27/68 (39.7%)	9/33 (27.3%)	0.22
Vancomycin	0/38 (0)	0/30 (0)	0/25 (0)	0/8 (0)	0/68 (0%)	0/33 (0%)	1
***Enterococcus faecalis***							
Vancomycin	0/14 (0)	2/19 (10.5)	0/7 (0)	0/4 (0)	2/33 (6.1%)	0/11 (0%)	1
***Enterococcus faecium***							
Vancomycin	1/15 (6.7)	0/8 (0)	3/11 (27.3)	0/2 (0)	1/22 (4.5%)	3/13 (23.1%)	0.13
***Gram negative***							
***Acinetobacter baumannii***							
Amikacin	6/9 (66.6)	10/15 (66.6)	32/35 (91.4)	15/17 (88.2)	16/24 (66.7%)	47/52 (90.4%)	0.019
Ceftazidime	8/9 (88.8)	13/15 (86.6)	34/35 (97.1)	17/17 (100)	21/24 (87.5%)	51/52 (98.1%)	0.09
Ciprofloxacin	6/9 (66.6)	11/15 (73.3)	34/35 (97.1)	17/17 (100)	17/24 (70.8%)	51/52 (98.1%)	0.001
Colistin	1/9 (11.1)	0/15 (0)	0/35 (0)	1/17 (5.8)	0/24 (0%)	1/52 (0%)	1
Gentamicin	4/9 (44.4)	10/15 (66.6)	16/35 (45.7)	12/17 (70.5)	14/24 (58.3%)	28/52 (53.8%)	0.71
Imipenem	4/9 (44.4)	8/15 (53.3)	34/35 (97.1)	17/17 (100)	12/24 (50%)	51/52 (98.1%)	<0.001
Piperacillin/Tazobactam	7/9 (77.7)	12/15 (80)	34/35 (97.1)	17/17 (100)	19/24 (79.2%)	51/52 (98.1%)	0.01
***Pseudomonas aeruginosa***							
Amikacin	15/32 (46.9)	15/33 (45.5)	17/25 (68)	4/11 (36.4)	30/65 (46.2%)	21/36 (58.3%)	0.24
Ceftazidime	18/32 (56.3)	19/33 (57.6)	21/25 (84)	8/11 (72.7)	37/65 (56.9%)	29/36 (80.6%)	0.016
Ciprofloxacin	20/32 (62.5)	14/33 (42.4)	17/25 (68)	5/11 (45.4)	34/65 (52.3%)	22/36 (61.1%)	0.39
Colistin	0/32 (0)	0/33 (0)	0/25 (0)	0/11 (0)	0/65 (0%)	0/36 (0%)	1
Imipenem	20/32 (62.5)	14/33 (42.4)	15/25 (60)	5/11 (45.4)	34/65 (52.3%)	20/36 (55.5%)	0.75
Piperacillin/Tazobactam	3/32 (9.4)	7/33 (21.2)	18/25 (72)	2/11 (18.2)	10/65 (15.4%)	20/36 (55.5%)	0.10
***Klebsiella pneumoniae***							
Ceftazidime	2/7 (28.6)	11/34 (32.4)	15/29 (51.7)	9/11 (81.8)	13/41 (31.7%)	24/40 (60%)	0.010
Ciprofloxacin	1/7 (14.3)	12/34 (35.3)	16/29 (55.2)	4/11 (36.4)	13/41 (31.7%)	20/40 (50%)	0.006
Meropenem	0/7 (0)	1/34 (2.9)	8/29 (27.6)	9/11 (81.8)	1/41 (2.4%)	17/40 (42.5%)	<0.001
Cefepime	0/7 (0)	11/34 (32.4)	15/29 (51.7)	9/11 (81.8)	11/41 (26.8%)	24/40 (60%)	<0.001
Cefoxitin	0/7 (0)	10/34 (29.4)	18/29 (62.1)	9/11 (81.8)	10/41 (24.4%)	27/40 (67.5%)	<0.001
Tobramycin	0/7 (0)	8/34 (23.5)	17/29 (58.6)	9/11 (81.8)	8/41 (19.5%)	26/40 (65%)	<0.001
Piperacillin/Tazobactam	0/7 (0)	8/34 (23.5)	15/29 (51.7)	9/11 (81.8)	8/41 (19.5%)	24/40 (60%)	<0.001
***Escherichia coli***							
Amikacin	0/35 (0)	0/57 (0)	4/47(8.5)	1/30(0)	0/92 (0%)	5/77 (6.5%)	0.05
Ciprofloxacin	7/35 (0.2)	3/57 (5.2)	4/47(8.5)	9/30(30)	10/92 (10.9%)	13/77 (16.9%)	0.006
Piperacillin/Tazobactam	1/35 (2.8)	1/57 (1.7)	4/47(8.5)	2/30(6.6)	2/92 (2.2%)	6/77 (7.8%)	0.37
Ceftazidime	2/35 (5.7)	0/57 (0)	4/47(8.5)	5/30(16.6)	2/92 (2.2%)	9/77 (11.7%)	0.02
Cefoxitin	4/35 (11.4)	1/57 (1.7)	4/47(8.5)	4/30(13.3)	5/92 (5.4%)	8/77 (10.4%)	0.18
Meropenem	1/35 (2.8)	0/57 (0)	1/47(2.1)	0/30(0)	1/92 (1.1%)	1/77 (1.3%)	0.52

* Data for the year 2005 refer to the period from January 2005 through June 2005.

** P-values refer to the comparison of proportions between 2002/2003 and 2004/2005.

**Table 4:** Data on antimicrobial resistance patterns and respective MIC_90 _of isolated bacteria.

	**2002**				**2003**				**2004**				**2005**			
***Microorganisms***	S*	I**	R***	**MIC**_**90**_^#^	**S**	**I**	**R**	**MIC**_**90**_	**S**	**I**	**R**	**MIC**_**90**_	**S**	**I**	**R**	**MIC**_**90**_

***Gram-positive***																
***Staphylococcus epidermidis***																
Oxacillin	31/202	0/202	171/202	4	46/261	0/261	215/261	4	36/214	0/214	179/214	4	15/94	0/94	75/94	4
Gentamicin	58/202	18/202	126/202	16	87/261	20/261	154/261	16	71/219	0/219	148/219	16	40/94	10/94	44/94	16
Vancomycin	202/202	0/202	0/202	2	261/261	0/261	0/261	2	219/219	0/219	0/219	2	94/94	0/94	0/94	4
***Staphylococcus aureus***																
Oxacillin	13/38	0/38	25/38	4	9/30	0/30	21/30	4	13/25	0/25	12/25	4	2/8	0/8	6/8	4
Gentamicin	16/38	13/38	9/38	8	21/30	5/30	4/30	8	19/25	0/25	6/25	4	5/8	1/8	2/8	0.5
Vancomycin	38/38	0/38	0/38	1	30/30	0/30	0/30	2	25/25	0/25	0/25	1	8/8	0/8	0/8	1
***Enterococcus faecalis***																
Vancomycin	14/14	0/14	0/14	2	17/19	0/19	2/19	2	6/6	0/6	0/6	2	4/4	0/4	0/4	2
***Enterococcus faecium***																
Vancomycin	13/14	0/14	1/14	1	8/8	0/8	0/8	1	2/2	0/2	0/2	32	2/2	0/2	0/2	1
***Gram negative***																
***Acinetobacter baumannii***																
Amikacin	3/9	1/9	5/9	64	5/15	0/15	10/15	64	3/35	2/35	30/35	64	2/17	2/17	13/17	64
Ceftazidime	1/9	2/9	6/9	64	2/15	1/15	12/15	64	1/35	0/35	34/35	64	0/17	0/17	17/17	64
Ciprofloxacin	3/9	0/9	6/9	4	3/15	0/15	12/15	4	1/35	0/35	34/35	4	0/17	0/17	17/17	4
Colistin	8/9	0/9	1/9	2	15/15	0/15	0/15	1	35/35	0/35	0/35	2	16/17	0/17	1/17	0.5
Gentamicin	5/9	0/9	4/9	16	5/15	5/15	5/15	16	19/35	6/35	10/35	16	5/17	4/17	8/17	16
Imipenem	5/9	2/9	2/9	16	6/15	4/15	5/15	16	1/35	5/35	29/35	16	0/17	4/17	13/17	16
Piperacillin/Tazobactam	2/9	1/9	6/9	128	4/15	1/15	10/15	128	1/35	1/35	33/35	128	0/17	3/17	14/17	128
***Pseudomonas aeruginosa***																
Amikacin	15/29	2/29	12/29	64	18/33	1/33	14/33	64	8/25	0/25	17/25	64	7/11	0/11	4/11	64
Ceftazidime	13/29	2/29	15/29	64	14/33	5/33	14/33	64	4/25	7/25	14/25	64	3/11	1/11	7/11	64
Ciprofloxacin	11/29	0/29	18/29	4	14/33	0/33	19/33	4	8/25	0/25	17/25	4	6/11	0/11	5/11	4
Colistin	29/29	0/29	0/29	2	33/33	0/33	0/33	2	25/25	0/25	0/25	2	11/11	0/11	0/11	2
Imipenem	11/29	14/29	4/29	16	19/33	9/33	5/33	16	10/25	6/25	9/25	16	6/11	4/11	1/11	16
Piperacillin/Tazobactam	26/29	0/29	3/29	64	26/33	1/33	6/33	128	7/25	11/25	7/25	128	9/11	0/11	2/11	128
***Klebsiella pneumoniae***																
Ceftazidime	5/7	0/7	2/7	64	23/34	0/34	11/34	64	14/29	1/29	14/29	64	2/11	0/11	9/11	64
Ciprofloxacin	6/7	0/7	1/7	1	22/34	1/34	11/34	4	13/29	0/29	16/29	4	7/11	0/11	4/11	4
Meropenem	7/7	0/7	0/7	0.25	33/34	0/34	1/34	0.5	21/29	4/29	4/29	8	2/11	1/11	8/11	16
Cefepime	7/7	0/7	0/7	2	23/34	1/34	10/34	64	14/29	7/29	8/29	64	2/11	0/11	9/11	64
Cefoxitin	7/7	0/7	0/7	4	24/34	0/34	10/34	64	11/29	0/29	18/29	64	2/11	0/11	9/11	64
Tobramycin	7/7	0/7	0/7	1	26/34	0/34	8/34	16	12/29	1/29	16/29	16	2/11	2/11	7/11	16
Piperacillin/Tazobactam	7/7	0/7	0/7	8	26/34	1/34	7/34	128	14/29	2/29	13/29	128	2/11	0/11	9/11	128
***Escherichia coli***																
Amikacin	35/35	0/35	0/35	2	57/57	0/57	0/57	2	43/47	4/47	0/47	4	29/30	0/30	1/30	4
Ciprofloxacin	28/35	0/35	7/35	4	54/57	0/57	3/57	0.5	43/47	0/47	4/47	4	21/30	0/30	9/30	4
Piperacillin/Tazobactam	34/35	0/35	1/35	4	56/57	1/57	0/57	4	43/47	1/47	3/47	8	28/30	1/30	1/30	4
Ceftazidime	33/35	0/35	2/35	2	57/57	0/57	0/57	1	43/47	3/47	1/47	1	25/30	0/30	5/30	2
Cefoxitin	31/35	1/35	3/35	16	56/57	1/57	0/57	4	43/47	4/47	0/47	4	26/30	1/30	3/30	16
Meropenem	34/35	1/35	0/35	0.25	57/57	0/57	0/57	0.25	46/47	0/47	1/47	0.25	30/30	0/30	0/30	2

Abbreviations: *S = susceptible, **I = intermediate, ***R = resistant, ^#^**MIC**_**90 **_= minimum inhibitory concentration for 90% of the corresponding microbial population

The antimicrobial susceptibility pattern of *Klebsiella pneumoniae *isolates changed significantly during our study. Increased resistance of *Klebsiella pneumoniae *isolates was noted for all beta lactams tested [specifically to piperacillin/tazobactam (p < 0.001), ceftazidime (p = 0.01), cefepime (p < 0.001), cefoxitin (p < 0.001) and meropenem (p < 0.001)] between the first and second period of the study. There was also increased resistance of *Klebsiella pneumoniae *to ciprofloxacin (p = 0.006) and tobramycin (p < 0.001).

Regarding the antimicrobial susceptibility pattern of Gram-positive bacteria during our study there was a considerable proportion of staphylococci with resistance against oxacillin (Table 1); however, the difference of the proportions of oxacillin resistant staphylococci between the two study periods was not statistically significant (Table 2). We did not isolate any staphylococci with resistance to vancomycin. *Enterococcus faecalis *and *Enterococcus faecium *were generally susceptible to vancomycin although some strains were resistant; however the difference of the proportions of VRE between the two study periods was not statistically significant (Table 2).

## Discussion

Patients with bacteremia have remained a challenge to treat. Knowledge of the hospital epidemiology and antimicrobial susceptibility pattern of blood isolates helps physicians to effectively manage blood stream infections. This is because considerable differences of the frequency of blood isolates are reported even from hospitals of similar size and mixture of patients of the same country [6].

In this study we evaluated the secular trends of the relative frequency and antimicrobial resistance of blood isolates in a newly founded Greek hospital. Gram-positive microorganisms are the most common blood isolates. Among them, coagulase negative staphylococci are the commonest blood isolates. The percentage of coagulase negative staphylococci (%) is higher in our study than that reported in large series (31.6%) [7-9]. It is possible that the proportion of coagulase negative staphylococci that were contaminants was considerable in our study. The interpretation of blood cultures that are positive for coagulase negative staphylococci has inherent difficulties and requires careful reasoning [10]. The observed relative frequency of MRSA was considerable high during the studied period. Data from the WHONET Greece (antimicrobial surveillance system) regarding the period from January 2005 through June 2005 showed that a significant proportion of *S. aureus *blood isolates are resistant to methicillin (MRSA strains). Specifically, 32.6%, 55.6%, and 69% of *S. aureus *blood isolates from medical wards, surgical wards, and ICUs respectively were MRSA.

In general, our results about the relative frequency of blood isolates in our newly founded hospital are not substantially different from those of hospitals that are functioning for a much longer period of time. Similar data have been reported in studies performed in hospitals elsewhere in Europe as well as in North America [7-9]. An explanation may be that it is not the microbial ecology of the structure (our newly founded tertiary urban hospital compared to hospitals that are functioning for longer time) but rather the characteristics of the admitted patients like comorbidity, medications, and other host factors that play the most important role in the relative frequency of blood isolates.

It is also noteworthy that the isolation of Candida spp from the blood was not uncommon during the study period. This is in agreement with the reports from all over the world regarding a considerable prevalence of fungemia due to extensive use of antibiotics, aggressive treatment of neoplastic disease, an expanding population of patients with AIDS with prolonged survivors, use of indwelling devices for ICU monitoring, and many other factors that predispose to fungal infections [8,9]. Although our hospital does not have a transplant unit, the observed high frequency of Candida isolates is probably explained by the fact that oncology patients and thus neutropenic patients constitute a significant portion of our patients.

We also evaluated in our study the trends of the antimicrobial resistance of the blood bacteria isolates in our newly founded hospital. The antimicrobial resistance of *Acinetobacter baumannii *showed an alarming increase during the study. *Acinetobacter baumannii *remained susceptible to colistin during the two periods, although the recovery of one resistant strain is of note [11,12]. Unfortunately, antimicrobial resistance increased also for *Klebsiella pneumoniae *for all of the 7 antibiotics it was tested for. These results are in concordance with data of the literature about the increasing antimicrobial resistance of Gram-negative bacteria [13-15]. In addition, it is noteworthy that the majority of bloodstream *K. pneumoniae *strains recovered in 2005 were resistant to meropenem, however this would probably reflect a nosocomial outbreak of a carbapenem-resistant *K. pneumoniae *clone.

We should acknowledge several limitations of our study. First, the results obtained from the Vitek II were confirmed by the E-test methodology only for colistin. Second, we did not proceed to the interpretation of the results of this study in terms of culture contamination or clinically relevant bloodstream infection. Third, pulsed-field gel electrophoresis was not performed to identify epidemic clones. Since molecular typing was not performed some of the studied isolates with antimicrobial resistance may be clonally related. Fourth, the number of patients visit the outpatient clinic of the hospital was not readily available. However, the number of positive blood cultures in the ambulatory outpatients is relatively small [16].

## Conclusion

Our data suggest that the relative frequency and the antimicrobial resistance pattern of the blood isolates in a newly founded hospital is not very different from those data described in the literature from other older hospitals around the world. In addition, an alarming increase of antimicrobial resistance was noted during our study for Gram-negative bacteria, especially *Acinetobacter baumannii *and *Klebsiella pneumoniae*.

## Competing interests

The author(s) declare that they have no competing interests.

## Authors' contributions

MEF conceived the idea for the study. SKK, PM, GG, DN, and PIF collected the data. MEF and PIF drafted the manuscript. All authors made revisions of the manuscript and approved its final version.

## Pre-publication history

The pre-publication history for this paper can be accessed here:


